# Ultra-Processed Food Consumption and Incidence of Obesity and Cardiometabolic Risk Factors in Adults: A Systematic Review of Prospective Studies

**DOI:** 10.3390/nu15112583

**Published:** 2023-05-31

**Authors:** Sara Paola Mambrini, Francesca Menichetti, Simone Ravella, Marta Pellizzari, Ramona De Amicis, Andrea Foppiani, Alberto Battezzati, Simona Bertoli, Alessandro Leone

**Affiliations:** 1 IRCCS Auxologico, Laboratory of Metabolic Research, San Giuseppe Hospital, 28824 Piancavallo, Italy; sara.mambrini@unimi.it; 2 International Center for the Assessment of Nutritional Status and the Development of Dietary Intervention Strategies (ICANS-DIS), Department of Food, Environmental and Nutritional Sciences (DeFENS), University of Milan, 20133 Milan, Italy; 3 IRCCS Auxologico, Laboratory of Nutrition and Obesity Research, Department of Endocrine and Metabolic Diseases, 20145 Milan, Italy; 4 IRCCS Auxologico, Clinical Nutrition Unit, Department of Endocrine and Metabolic Diseases, 20145 Milan, Italy

**Keywords:** ultra-processed foods, NOVA, obesity, cardiometabolic risk, adults, cohort study systematic review

## Abstract

Ultra-processed foods (UPF) are energy-dense, nutritionally unbalanced products, low in fiber but high in saturated fat, salt, and sugar. Recently, UPF consumption has increased likewise the incidence of obesity and cardiometabolic diseases. To highlight a possible relationship, we conducted a systematic review of prospective studies from PubMed and Web of Science investigating the association between UPF consumption and the incidence of obesity and cardiometabolic risk factors. Seventeen studies were selected. Eight evaluated the incidence of general and abdominal obesity, one the incidence of impaired fasting blood glucose, four the incidence of diabetes, two the incidence of dyslipidemia, and only one the incidence of metabolic syndrome. Studies’ quality was assessed according to the Critical Appraisal Checklist for cohort studies proposed by the Joanna Briggs Institute. Substantial agreement emerged among the studies in defining UPF consumption as being associated with the incident risk of general and abdominal obesity. More limited was the evidence on cardiometabolic risk. Nevertheless, most studies reported that UPF consumption as being associated with an increased risk of hypertension, diabetes, and dyslipidemia. In conclusion, evidence supports the existence of a relationship between UPF consumption and the incidence of obesity and cardiometabolic risk. However, further longitudinal studies considering diet quality and changes over time are needed.

## 1. Introduction

Obesity is a growing worldwide health problem. It is characterized by excessive adiposity that can compromise health status. According to the World Health Organization, obesity affects more than 1 billion people worldwide, 650 million of whom are adults [1]. Obesity is closely linked with metabolic syndrome [2], defined by the National Institutes of Health as a cluster of interconnected metabolic abnormalities, including central adiposity, dyslipidemia, high blood pressure, and impaired fasting glucose [3]. Both obesity and metabolic syndrome are associated with increased risk for mortality and many non-communicable diseases (NCDs) [2].

Obesity and metabolic syndrome are complex, multifactorial diseases whose causes are not yet fully elucidated. However, it is well known that dietary habits play a crucial role in influencing cardiometabolic risk [4]. Several epidemiological studies support an inverse association between adherence to healthy dietary patterns, such as the Mediterranean diet, and cardiometabolic risk [5,6,7]. In contrast, a diet rich in highly processed foods is strongly associated with obesity and related metabolic comorbidities [8,9,10].

The NOVA food system was proposed in 2010 to classify foods according to the level of processing [11]. According to this system, foods are classified into four different food groups according to the type, extent, and scope of industrial processes to which foods have been subjected. The first group refers to unprocessed or minimally processed foods. This group includes edible parts of plants or animals and natural foods altered by processes aimed to make them edible, suitable for preservation, safe, or more palatable. The second group refers to processed culinary ingredients including lard, butter, oils, salt, and sugar. They are generally used in combination with foods to make meals and dishes more palatable. The third group refers to processed foods. This group includes food products obtained by adding substances from group 2 to group 1 foods in order to increase their shelf life and enhance sensory qualities. They mostly contain two or three ingredients. The last group references ultra-processed foods (UPF). This group includes formulations mainly made of unmodified and modified substances extracted from foods and assembled with few, if any, whole foods. They also contain food additives to increase palatability, sensory characteristics, and shelf-life. They generally contain five or more ingredients. Examples of UPFs are breakfast cereals, packaged savory and sweet snacks, packaged bread, margarine, reconstituted meat products, pre-prepared frozen dishes, instant soups, sweet and carbonated beverages, and distilled alcoholic beverages.

Several studies reported that UPF consumption is rising, accounting now for more than half of the daily calories of US [12], Canadian [13], or British [14] diets. Moreover, it has been shown that high UPF consumption leads to a nutritionally unbalanced diet, rich in energy, saturated fat, sugar, and salt and poor in fiber, vitamins, and minerals [14], potentially affecting the risk for obesity and cardiometabolic risk factors [15]. Therefore, we conducted a systematic review aimed to summarize the available literature on the association between UPF consumption and the incidences of obesity and cardiometabolic risk factors among adults.

## 2. Materials and Methods

### 2.1. Search Strategy

Preferred Reporting Items for Systematic Reviews and Meta-Analysis guidelines (PRISMA) were followed to carry out the study [7]. Studies included in the present review were identified by searching in two electronic databases, including PubMed and Web of Science, using the following search string: (ultraprocessed food* OR ultra-processed food* OR ultra processed food* OR NOVA food*) AND (obesity OR overweight OR waist circumference OR blood pressure OR hypertension OR dyslipidemia OR triglycerides OR cholesterol OR impaired fasting glucose OR diabetes OR metabolic syndrome OR cardiovascular disease OR cardiovascular risk). Electronic search was carried out in September 2022. This systematic review was registered in PROSPERO with registration number CRD42023423112.

### 2.2. Study Selection, Inclusion and Exclusion Criteria

Initially, we proceeded to exclude duplicates. Then, two independent investigators (S.P.M. and S.R.) selected articles based on title and abstract. The selected articles were then evaluated for eligibility. To be included in the present review, articles needed to be original, include healthy participants aged 18 years or older, written in English, have a prospective cohort study design, use NOVA classification to define UPF, and have as outcomes general or central obesity and cardiometabolic risk factors. No country/region/ethnicity nor date restrictions were applied. Cross-sectional and case-control studies were excluded. Studies limiting the evaluation only to a specific food category included in the definition of UPF, such as reconstituted meat products or sugar-sweetened beverages, or that assess household availability or purchase of UPF were excluded. We further excluded meta-analyses, review articles, congress abstracts, letters, and comments. Disagreements in study selection were resolved through consensus or by seeking the opinion of a third investigator (A.L.) if consensus could not be reached.

### 2.3. Data Extraction

From each article, we extracted the following data: main author, country, year of publication, number of participants, outcomes, dietary assessment method, confounding factors, and main results. Two independent investigators (S.P.M. and F.M.) reviewed selected articles and performed data extraction. A third investigator (A.L.) supervised data extraction and solved inconsistencies and disagreements.

### 2.4. Quality Assessment

Two independent investigators (S.P.M. and M.P.) conducted the quality assessment. The Critical Appraisal Checklist for cohort studies proposed by Joanna Briggs Institute was used to assess the methodological quality of the selected studies [16]. The checklist included 11 items related to the following critical domains: population characteristics, follow-up, outcomes, exposure, confounders, and statistical analysis. For each item, it was possible to respond with “no”, “yes”, “unclear”, or “not applicable”. Based on the responses, an overall critical assessment of the quality of the study was obtained. In cases where the two investigators disagreed in answering individual items, the opinion of a third investigator (A.L.) was sought. Studies that received a positive score in at least half of the items were considered to be of acceptable quality for inclusion in this Review [9].

## 3. Results

**Figure 1:**
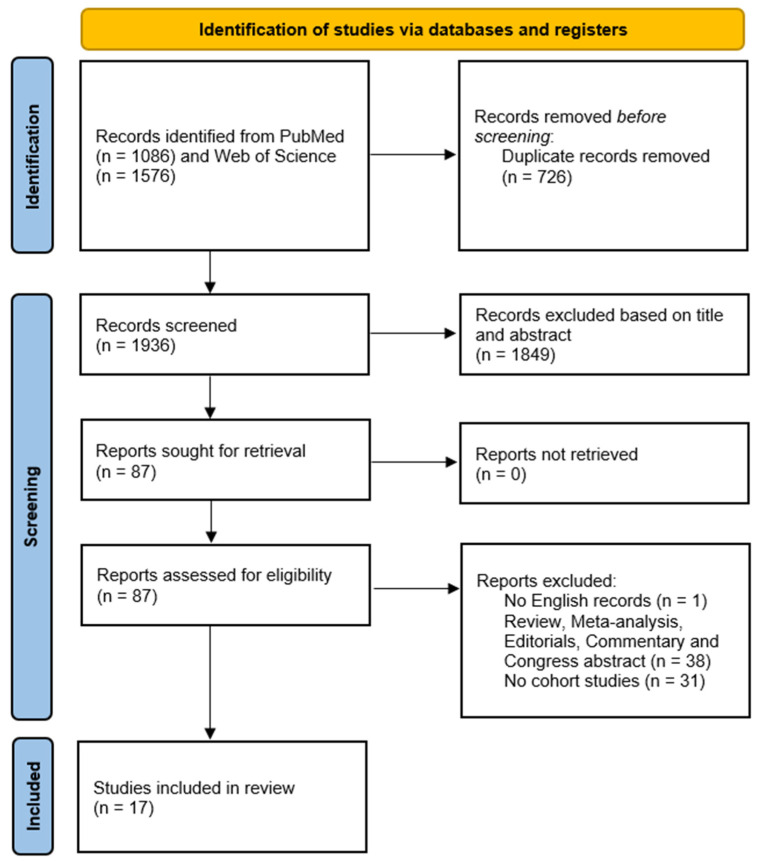
Flow chart of studies’ selection process.

**Figure 2:**
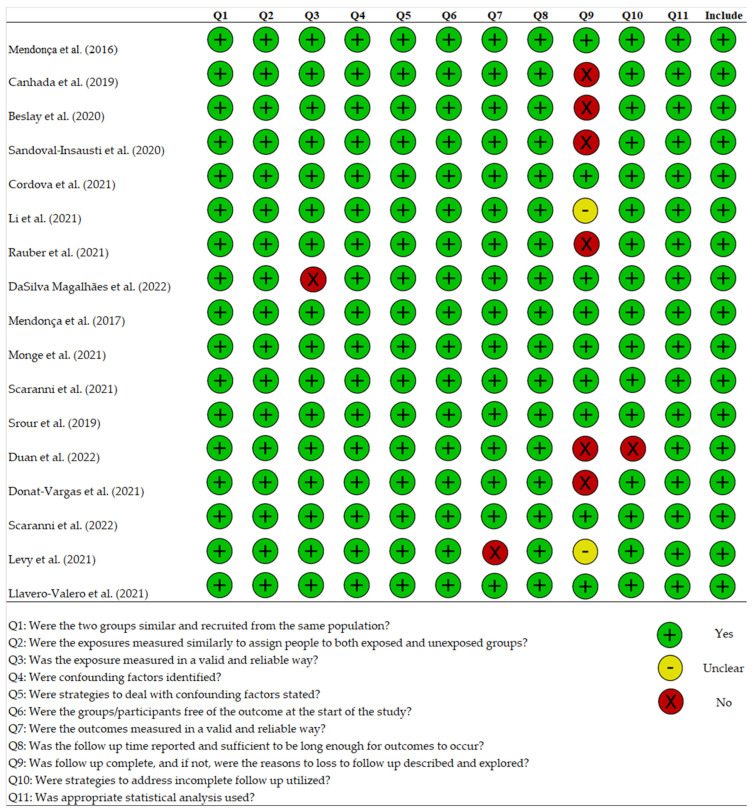
Quality checklist. Studies receiving a positive score in at least half of the items were considered to be of acceptable quality for inclusion [17,18,19,20,21,22,23,24,25,26,27,28,29,30,31,32,33].

A total of 2662 articles were initially found on Pubmed and Web of Science (Figure 1). We then removed 717 duplicates and discarded an additional 1852 articles based on title and/or abstract, as they were deemed irrelevant to the review. The remaining 93 records were evaluated for eligibility. Of these, 2 articles were not written in English, 42 were review, meta-analysis, editorial, commentary, or congress abstracts, and 32 were original studies but with a study design different from the cohort study (mainly cross-sectional), and therefore were removed. At the end of the evaluation process, 17 studies were included in this systematic review. The quality assessment of the selected studies is shown in Figure 2.

### 3.1. Study Characteristics

The 17 studies included a total of 822,213 adults of both sexes (Table 1). The sample size ranged from a minimum of 652 to a maximum of 348748. Four studies were conducted in Brazil [17,18,19,20], two in France [21,22] one in Mexico [23], one in the Netherlands [24], five in Spain [25,26,27,28,29], two in the UK [30,31], and another in China [32]. One study used data from the EPIC study cohort, which collects data from several European countries such as Denmark, France, Germany, Italy, and Norway [33]. Regarding dietary assessment, nine studies used food frequency questionnaires (FFQ) consisting of a different number of questions [18,19,20,23,24,26,27,28,33], six studies used the 24 h recall [17,21,22,30,31,32], and two studies used dietary history [25,29]. The cohort study published by Cordova et al. uses both Food Frequency Questionnaires (FFQ) and dietary interviews to collect data on UPF consumption. UPF consumption (exposure variable) was assessed as the percentage of energy from UPF (%UPF_energy_) in six studies [17,18,23,25,29,31], as servings of UPF consumed per day in two studies [26,27], as grams of UPF (UPF_g_/day) per day in five studies [19,22,28,32,33] and as the proportion of UPF intake in the total weight of food consumed (%UPF_intake_) in three studies [21,30]. In one study, UPF consumption was expressed both as a %UPF_energy_ and a %UPF_intake_ [20].

### 3.2. Consumption of Ultra-Processed Food, Excess Body Weight, and Abdominal Obesity

Eight cohort studies investigated the relationship between UPF consumption and the risk of weight excess and abdominal obesity, all finding a positive relationship [17,20,21,25,26,32,33,34]. Four studies focused on the risk of overweight and obesity [17,21,26,33] and two studies on the risk of abdominal obesity [20,25,31], while two other studies investigated both the risk of overweight and obesity and of abdominal obesity [31,32]. Mendonca et al. [26] analyzed data from the SUN cohort, reporting that normal-weight participants consuming higher amounts of UPF, expressed as consumption of servings per day, had a 26% higher risk of developing overweight or obesity during follow-up (HR = 1.26; 95% CI: 1.10, 1.45, *P*_trend_ = 0.001), than participants with lower UPF consumption. Similarly, data from the ELSA cohort [17] showed that normal-weight participants in the uppermost quartile of UPF consumption had a 20% higher risk of overweight and obesity during follow-up than participants in the lowest quartile (RR = 1.03, 95% CI: 1.0, 1.40). However, no association between UPF consumption and incident risk of obesity was found among participants who were overweight at baseline (fourth vs. first quartile RR = 1.02, 95% CI: 0.85, 1.21). Results from the French NutriNet-Santè cohort [21], including 110260 adults, reported an 11% increase in the risk of developing overweight or obesity among normal-weight participants (HR = 1.11, 95% CI: 1.08, 1.14; *p* < 0.001) and a 9% increase in the risk of developing obesity among overweight participants (HR = 1.09, 95% CI: 1.05, 1.13; *p* < 0.13), associated with a 10% increase in the % of energy from UPF. Data from the EPIC cohort [33], including a multi-national population of 348748 adults, also reported that normal-weight participants in the fifth quintile of UPF consumption had a 15% higher risk of developing overweight or obesity (RR = 1.15, 95% CI: 1.11, 1.19; *P*_trend_ < 0.001) than participants in the first quintile of UPF consumption. Similarly, overweight participants in the highest quantile of UPF consumption had a 16% higher risk of developing obesity (RR = 1.16, 95% CI: 1.09, 1.23; *P*_trend_ < 0.001) than overweight participants with low consumption of UPF. Data from the China Health and Nutrition Survey (CHNS) [32], including 12451 adults of both sexes, showed a higher risk of overweight and obesity (OR = 1.45, 95% CI: 1.21, 1.74) and abdominal obesity (OR = 1.50, 95% CI: 1.29, 1.74) in participants consuming ≥50 g/day of UPF than non-consumers. Additionally, Rauber et al. [34] found that participants in the fourth quartile of UPF consumption presented a 79% and 30% greater risk of developing obesity (HR = 1.79, 95% CI: 1.06, 3.03) and abdominal obesity (HR = 1.30, 95% CI: 1.14, 1.48), respectively, than participants in the first quartile of UPF consumption. Sandoval et al. [25] reported that, in the Seniors-ENRICA-1 cohort, the incidence of abdominal obesity in elders was significantly higher in participants in the uppermost tertile of UPF consumption than participants in the lowest one (OR = 1.61; 95% CI: 1.01, 2.56; *P*_trend_ = 0.048). Finally, DaSilva Magalhães et al. [20] assessed UPF consumption in 896 men and women aged 23–25 years and related it to the incidence of metabolic syndrome and its components at ages 37–39. They found that UPF consumption was associated with a higher risk of abdominal obesity in women (RR = 1.01, 95% CI: 1.00, 1.02) but not in men.

### 3.3. Consumption of Ultra-Processed Food, Impaired Fasting Glucose, and Diabetes Mellitus

The association between UPF consumption and incident risk of impaired fasting glucose was investigated in only one study [20]. On the other hand, four studies focused on the relationship between UPF consumption and the risk of type 2 diabetes (T2D) [24,28,30,35]. New cases of diabetes were identified by self-reported data supported by medical records [20,28,35] or nurse interviews [30] or blood glucose and HbA1c measurements [24]. Silva Magalhães et al. [20] reported that UPF consumption at 23–25 years was not associated with impaired fasting glucose at 37–39 years (%UPF_energy_ RR = 1.00, 95% CI: 0.99, 1.01; %UPF_intake_ RR = 0.99; 95% CI: 0.98, 1.00). Concerning the incident risk of T2D, in the NutriNet-Santé cohort, Srour et al. [35] found a 15% higher risk of T2D associated with an increment of 10% of UPF consumption (grams per day) (HR = 1.15, 95% CI, 1.06–1.25; *p* = 0.001). Similarly, for each 100g/d increment in the absolute amount of UPF, the risk of T2D increased by 5% (HR = 1.05; 95% CI: 1.02, 1.08). In the Lifelines cohort, including participants aged 35–70 years, Duan et al. [24] found that a 10% increment in UPF consumption was associated with a 25% higher risk of T2D (OR = 1.25, 95% CI: 1.16, 1.34). Levy et al. [30], in the UK Biobank cohort, found that participants in the fourth quartile of UPF consumption had a 44% higher risk of T2D than participants in the first quartile of UPF consumption (HR = 1.44; 95% CI: 1.04, 2.02). Moreover, they observed a significant 12% increased risk of T2D per 10%-point increments in UPF consumption (HR = 1.12; 95% CI: 1.04, 1.20). Finally, in the SUN cohort, Llavero-Valero et al. [28] found a 53% increased risk of T2D (HR = 1.53; 95% CI: 1.06, 2.22; *P*_trend_ < 0.001) in participants in the third tertile of UPF consumption as compared with participants in the first one.

### 3.4. Consumption of Ultra-Processed Food and Hypertension

Four studies focused on the relationship between UPF consumption and the incidence of hypertension [18,20,23,27]. Three studies [18,20,27] evaluated this relation both in men and women, whereas only one [23] did so for women. Additionally, two studies evaluated the outcome as self-reported diagnoses of hypertension [23,27] while in the other two [18,20], the outcome was defined by measuring the systolic and diastolic blood pressure during the follow-up. Mendonça et al. [27] observed a 21% higher risk of hypertension among participants in the uppermost tertile of UPF consumption compared with participants in the first tertile (HR = 1.21, 95% CI: 1.06, 1.37, *P*_trend_ = 0.004). Similarly, Scaranni et al. [18] found participants of the ELSA-Brasil cohort with high UPF consumption to have a 17% increased risk of developing hypertension (OR = 1.17; 95% CI: 1.00, 1.37) than participants with low UPF consumption. In contrast, in the Mexican Teachers’ Cohort (MTC), including 64934 women, Monge et al. [23] did not find UPF consumption significantly associated with the incident risk of hypertension when comparing extreme categories of UPF consumption (IRR = 0.96; 95% CI: 0.79, 1.16; *P*_trend_ = 0.95). Finally, DaSilva et al. [20] reported that the %UPF at 23–25 years is marginally associated with the risk of hypertension at 37–39 years old (%kcal adjusted RR = 1.01; 95% CI: 1.00, 1.02).

### 3.5. Consumption of Ultra-Processed Food and Lipid Profile

Among the studies selected, three studies investigated the association between UPF consumption and the incidence of dyslipidemia [19,20,29]. Two of them focused on adults [19,20] and the other one on elders (≥60 years old) [29]. Of the 1821 participants from the Seniors-ENRICA cohort, Donat-Vargas et al. [29] reported that participants in the third tertile of energy intake of UPF had a higher risk for hypertriglyceridemia (OR = 2.66; 95% CI: 1.20, 5.90; *P*_trend_ = 0.011) and low HDL cholesterol (OR = 2.23; 95% CI: 1.22, 4.05; *P*_trend_ = 0.012) than participants in the first tertile. No association between UPF consumption and high LDL cholesterol emerged. Scaranni et al. [18,19], in the ELSA-Brasil cohort, observed that participants with medium and high UPF consumption had a higher risk of developing isolated hypertriglyceridemia (OR = 1.14, 95% CI: 1.03 and 1.26; OR = 1.30, 95% CI: 1.17 and 1.45), isolated hypercholesterolemia (OR = 1.12, 95% CI: 1.00 and 1.27; OR = 1.28, 95% CI: 1.12 and 1.47), low HDL cholesterol (OR = 1.12, 95% CI: 1.00 and 1.24; OR = 1.18, 95% CI: 1.05 and 1.32), and mixed hyperlipidemia (OR = 1.21, 95% CI: 1.05 and 1.39; OR = 1.38, 95% CI: 1.18 and 1.62) than participants consuming lower amounts of UPF. However, the association with low HDL cholesterol was lost when BMI was included in the model. On the contrary, DaSilva et al. [20] reported that UPF consumption at 23–25 years old was not associated with the risk of hypertriglyceridemia at the age of 37–39. On the other hand, UPF was associated with a higher risk of low HDL only in women (RR = 1.02, 95% CI: 1.01, 1.04).

### 3.6. Consumption of Ultra-Processed Food and Metabolic Syndrome

Only one study evaluated the relationship between UPF consumption and the incident risk of MetS [20]. The authors [20] reported that UPF consumption at 23–25 years was not associated with the risk of MetS at 37–39 years (RR = 1.00; 95% CI: 0.99, 1.01).

## 4. Discussion

In this systematic review, we summarized all available prospective studies focused on the association between UPF consumption and the incidence of obesity and cardiometabolic risk factors in adults. All studies included reported UPF consumption associated with the risk of developing overweight and obesity [17,21,26,31,32,33]. Moreover, although more limited in number, the studies included in this review agreed on the association between UPF consumption and abdominal obesity [17,20,25]. Much more limited and heterogeneous were the prospective studies investigating the association between UPF consumption and cardiometabolic risk factors. However, most evidence supports the existence of a relationship with an increased risk of dyslipidemia, hypertension, and diabetes.

Traditionally, UPFs are energy-dense products with low nutritional quality. They contribute to increasing dietary intakes of saturated and trans fatty acids, sugars, refined carbohydrates, and sodium, and to reducing dietary intakes of fiber, micronutrients, and other protective bioactive compounds naturally present in foods [14]. In addition, it has been reported that these products are less satiating and characterized by a greater glycemic response than minimally processed foods [36]. Because of the higher energy density, low satiating effect, and large portion packing [37], consumption of these products may promote excess energy intake [38]. The minimal preparation skills required for UPF consumption can then alter eating patterns, leading to the rapid and unconscious consumption of food while engaged in routine alternative activities [39,40], altering neural and digestive functions that signal hunger and satiety, leading to overconsumption [41,42]. In addition, given their high fat and sugar contents, they can alter the reward neurocircuit mechanism, leading to increased food cravings and further exacerbating overconsumption [43,44]. To this it should be added that, under conditions of energy excess, the increased glucose response induced by UPF consumption may alter the insulin response, favoring the storage of excess nutrients in adipose tissue rather than their oxidation [45]. Excessive energy intake and obesity resulting from UPF consumption are certainly reasons for the development of cardiometabolic risk factors. However, this cannot entirely explain the associations observed between UPF and cardiometabolic risk factors, as many studies controlled their models for BMI and total energy intake. Many UPFs, such as condiments, broths, soup powders, and processed meats, have high levels of salt, contributing to higher sodium intake, a known risk factor for developing hypertension [46]. Added sugar could also alter fructose metabolism in the liver, promoting insulin resistance in the liver and throughout the body. Added fructose has been found to contribute to low-grade inflammation and oxidative stress, potentially causing β-cell damage and reducing insulin secretion [47]. Moreover, excess dietary fructose has been reported to impair the catabolism of very low-density lipoprotein cholesterol (VLDL-C) and increase VLDL-C synthesis, leading to an increase in triglycerides [48]. UPFs are also a source of trans and saturated fatty acids, which may contribute to an increased risk of dyslipidemia. Several RCTs found trans fatty acids having adverse effects on lipid profile [49], such as decreasing HDL cholesterol [50]. In addition, the intake of saturated fatty acids may have a negative impact on lipid metabolism, especially by virtue of the fact that UPFs are simultaneously low in PUFA [51]. Finally, UPFs contain plenty of chemical additives, synthetic antioxidants, and preservatives; many of these have been shown to increase the risk of obesity, deteriorate the lipid [52] and glucose profiles [53], and induce low-grade inflammation and metabolic syndrome [54]. In addition, the packaging of UPF can release known endocrine-disrupting chemicals (e.g., bisphenol A) into the food, increasing the risk of obesity and cardiometabolic risk [55,56,57]. Finally, it is presumed that those who consume high amounts of UPF have lower consumption of whole grains, fruits, and vegetables, limiting the intake [58] of micronutrients and bioactive compounds that may reduce cardiometabolic risk.

Despite the associations found, some considerations need to be made to evaluate the associations between UPF consumption and the incidence of obesity and cardiometabolic risk factors and to compare the results between studies. Only six studies controlled for dietary patterns or quality [19,21,22,24,25,33]. Considering the overall dietary pattern avoids potential confounding by other aspects of the diet, allows for evaluation of the interaction between synergistic components, and increases the ability to assess stronger effects due to the cumulative effects of many dietary characteristics [59]. An approach focused only on UPF consumption does not take into account the substitution effects of foods and associated foods [60]. Consumption of UPFs in a varied and balanced diet may not have the same effect as when they are consumed in a high-calorie diet, in which consumption of UPFs leads to the reduction of foods of higher nutritional value [9]. In addition, a very limited number of studies have repeatedly measured exposure. Dietary habits may change over time according to the food offered and living or environmental conditions, and consequently, they may influence the risk of obesity and cardiometabolic risk factors. An additional source of bias may be the method used for diet assessment. Most studies used 24 h recall and FFQ, while only two studies used dietary history. Although they are all accepted methods for evaluating dietary consumption, they are susceptible to recall bias and to difficulties in quantifying portions, compared with prospective methods based on recording and weighing foods consumed. Moreover, although a single 24 h recall is generally considered valid for assessing a population’s food intake, to have a better estimation of habitual UPF consumption, especially given the wide range of products that are part of it, it may be necessary to consider multiple food days. In addition, although the FFQ is a commonly used method to assess the diet–health association, it suffers from some measurement errors. The dietary assessment is often limited to a specific list of foods that varies according to the questionnaire used and the quantification of intake is not as accurate as with the 24 h recall or food diary [61]. Moreover, it should be remembered that all of these methods are not specifically designed to assess UPF consumption as it is defined by the NOVA classification. This can determine an overestimation or underestimation of UPF consumption. Finally, the use of different units of measurement to assess exposure to UPF (e.g., %UPF_energy_, servings, g/day, (%UPF_intake_) may have contributed to increased heterogeneity among studies. Future studies should therefore standardize the units of measurement to facilitate the comparison of results. Since obesity, as well as cardiometabolic risk and other NCDs, is strongly related to caloric intake, it is important to discern the effect of UPFs from that of total energy intake. Using a nutrient density model (%UPFenergy), without further adjustment for total calories, is not sufficient to remove the effect of total energy intake [62]. In addition, this approach does not allow for the consideration of UPFs that do not provide energy (e.g., artificially sweetened beverages). Similarly, the use of daily consumption frequency alone, without portion quantification, does not allow for true quantification of the foods consumed (e.g., many small portions might be equivalent to one large portion). These limitations can be overcome by using the total weight of foods consumed. In addition, using energy-adjusted food weight with the residual method would control for confounding factors by total energy intake and remove extraneous variations due to total energy intake [62].

Among the limitations of the present review is that many of the studies assessed exposure only at baseline. It must be considered that dietary habits assessed at baseline may have changed during follow-up, affecting risk estimates. To obtain a better representation of dietary habits and identify the direction of their relationship with cardiometabolic risk, more longitudinal studies with repeated assessments of food intake are needed. Moreover, several studies had a retention rate during follow-up that was potentially suboptimal (<80%). In addition, although the adjustment for confounders was considered satisfactory overall, several studies did not consider diet quality, which may have influenced the result. Poor geographic representativeness is a further limitation. The majority of the studies were conducted in Brazil, Spain, France, and England, limiting the generalizability of the results for other countries. Although the NOVA classification is internationally recognized, it may not be appropriate in all countries due to different cultural and dietary habits, as well as different industrial food production technology. For example, it was found that 23% of UPFs sold in Italy were of high nutritional quality considering three front-of-pack labeling schemes [63]. Therefore, further studies need to be conducted on other populations in order to develop correct nutrition policies and recommendations.

Nevertheless, this systematic review also has some strengths. We included only prospective cohort studies that, by measuring events in a temporal sequence, allow us to distinguish causes from effects [64]. This also made it possible to limit the variability due to the use of different study designs. In addition, we only included studies that used the NOVA classification, limiting the variability among studies due to different methods of defining UPF.

## 5. Conclusions

In conclusion, studies currently available in the literature agree that the consumption of ultra-processed foods is associated with the incidence of obesity. Less clear is its relationship with the incidence of outcomes related to cardiometabolic risk. Despite the positive associations found between the consumption of ultra-processed foods and cardiometabolic risk, the studies reported in the literature are still very limited, especially for some outcomes, and some results are conflicting, probably due to the adoption of different methods for assessing dietary habits, adjustment for possible confounders not always optimal, and other methodological limitations. Further longitudinal studies are therefore needed to better compare these associations, possibly considering overall dietary quality and dietary changes over time.

## Figures and Tables

**Table 1:** Summary of the selected studies investigating the association between UPF consumption and obesity and cardiometabolic risk factors in adults.

Author (Year)	Country (Cohort)	Subjects (n) and Baseline Characteristics	Outcome	Follow-Up Time	Dietary Assessment	Covariates Included in the Fully Adjusted Model	Type of Exposure	Results
Mendonça et al. (2016) [26]	Spain (SUN cohort)	8451 participants 35.1% men 64.9% women Age: 37.6 ± 11.0 years	Overweight/obesity	Median follow-up: 8.9 years	Semi-quantitative FFQ (136 items)	Sex, age, baseline BMI, educational status, marital status, physical activity, smoking status, siesta sleep, television watching, following a special diet at baseline, snacking between meals, and consumption of fruit and vegetables.	servings/d	Participants in the fourth quartile of UPF consumption had a higher risk of developing overweight or obesity (HR = 1.26, 95% CI: 1.10, 1.45, *P*_trend_ = 0.001) than participants in the first quartile.
Canhada et al. (2019) [17]	Brazil (ELSA cohort)	11,827 participants 45% men 55% women Age: 51.3 ± 8.7 years	Overweight/obesity	Mean follow-up: 3.8 years	FFQ (114 items)	Age, sex, school achievement, center, and color/race, as well as smoking and physical activity, waist/weight gain, incidence of overweight/obesity, baseline BMI, and baseline waist circumference.	%UPF_energy_	Participants in the fourth quartile of UPF consumption (>30.8 %) presented 20% greater risk (RR:1.20; 95% CI: 1.03, 1–40) of incident overweight and obesity than participants in the first quartile (<17.8%). No association between UPF quartiles and risk of incident obesity among overweight participants was observed (RR:1.02; 95% CI: 0.85, 1.21).
Beslay et al. (2020) [21]	France (French NutriNet-Santè cohort)	110,260 participants 22.8% men 78.2% women Age: 43.1 ± 14.6 years	Overweight/obesity	Median follow-up: 4.1 years	24 h dietary record	Age, sex, marital status, BMI, educational level, physical activity, smoking status, alcohol intake, number of 24 h dietary records, energy intake, health, and Western dietary pattern.	%UPF_intake_	Normal-weight participants with low UPF consumption had a lower risk of developing overweight or obesity during follow-up (HR_Q4 vs.Q1_ = 1.22, 95% CI: 1.14, 1.31, *P*_trend_ < 0.001) than those with a higher intake. Moreover, a 10% increment of UPF intake was associated with a higher risk of developing overweight or obesity (HR = 1.10, 95% CI: 1.07, 1.13; *P* < 0.001). Non-obese subjects with low UPF consumption had a lower risk of developing obesity during follow-up (HR_Q4 vs.Q1_ = 1.20, 95% CI: 1.08, 1.33, *P*_trend_ < 0.001) than those with a higher intake. Moreover, a 10% increment of UPFs intake was associated with a higher risk of developing obesity (HR = 1.11, 95% CI: 1.07, 1.15; *P* < 0.001).
Sandoval-Insausti et al. (2020) [25]	Spain (Seniors-ENRICA-1)	652 participants 55.7% men 44.3% women Age: 67.08 ± 5.8 years	Abdominal obesity	Median follow-up: 6 years	Dietary history (DH-ENRICA) record	Age, sex, educational level, marital status, ex-drinker status, smoking, physical activity in the household, physical activity during leisure time, prevalence of chronic disease, number of medications consumed daily, and adherence to Mediterranean diet.	%UPF_energy_	Participants in the first tertiles of UPF consumption had a higher risk of developing abdominal obesity (RR: 1.61; 95% CI: 1.01, 2.56, *P*_trend_=0.048) than participants in the first tertile.
Cordova et al. (2021) [33]	Denmark, Germany, Italy, France, Greece, the Netherlands, Spain, Norway, Sweden and the UK (EPIC cohort)	348,748 participants 26.6% men 73.4% women Age: 51.7 ± 9.0 years	Overweight/obesity	Median follow-up: 5 years	(a) Quantitative FFQ (Italy, Spain, the Netherlands, Germany, and France) (b) Semi-quantitative FFQ (Denmark, Naples (Italy), Norway, and Umeå (Sweden), (c) A combination of semi-quantitative FFQ and 7- and 14-day records in the UK and Malmo (Sweden).	Age, sex, BMI baseline, education level, smoking history, physical activity, alcohol intake, Mediterranean diet score, and plausibility of dietary energy reporting.	g/day	Normal-weight participants in the fifth quintile of UPF consumption had a 15% higher risk (RR = 1.15, 95% CI: 1.11, 1.19, *P*_trend_ <0.001) of becoming overweight or obese during follow-up than participants in the first quintile. Similarly, participants with overweight in the highest quintile of UPF consumption had a 16% higher risk (RR = 1.16; 95% CI: 1.09, 1.23, *P*_trend_ <0.001) of becoming obese during follow-up than participants in the lowest quintile.
Li et al. (2021) [32]	China (CNHS cohort)	12,451 participants 48.7% men 51.3% women Age: 43.7 ± 14.7 years	Overweight/obesity and abdominal obesity	10 years	3-day 24 h dietary recall	Age, sex, income, urbanization, education, smoking, alcohol drinking, and physical activity, energy intake, fat intake, and dietary patterns.	g/day	Participants consuming 1–19 g/day, 20–49 g/day, or ≥ 50 g/day of UPF were at a higher risk of developing overweight and obesity and abdominal obesity than non-consumers. Adjusted ORs for overweight and obesity were 1.45 (95% CI: 1.26, 1.65), 1.34 (95% CI: 1.15–1.57), and 1.45 (95% CI: 1.21–1.74), respectively. Adjusted ORs for abdominal obesity were 1.54 (95% CI: 1.38, 1.72), 1.35 (95% CI: 1.19, 1.54), and 1.50 (95% CI: 1.29, 1.74), respectively.
Rauber et al. (2021) [31]	England, Scotland and Wales (UK Biobank)	22,659 participants 47.9% men 52.1% women Age: 55.9 ± 7.4 years	General and abdominal obesity	Median follow-up: 5 years	24 h dietary recall	Sex, BMI, waist circumference or body fat at baseline, smoking status, level of physical activity, sleep duration, Index of Multiple Deprivation (IMD).	%UPF_energy_	Non-obese participants in the uppermost quartile of UPF consumption were at a higher risk of developing obesity (HR = 1.79, 95% CI: 1.06, 3.03) than participants in the lowest quartile. Similarly, participants with normal waist circumference at baseline but in the first quartile of UPF consumption were at a higher risk of developing abdominal obesity (HR = 1.30, 95% CI: 1.14, 1.48) than participants in the lowest quartile.
DaSilva Magalhães et al. (2022) [20]	Brazil (Ribeirão Preto cohort)	896 particpants 44.3% men 55.7% women Age: 23–25 years	MetS and its components	14–16 years	Semi-quantitative FFQ (83 items)	Sex, age, education, marital status, skin color, family income, smoking, level of physical activity, and alcohol consumption. In the analyses with the consumption of UPF in %g, total energy intake was additionally included.	%UPF_energy_ and %UPF_intake_	UPF consumption was not associated with the risk of metabolic syndrome (%kcal PR: 1.00; 95% CI: 0.99–1.01; %g PR: 1.00; 95% CI: 0.99–1.01). However, women with higher UPF consumption were at a higher risk of developing abdominal obesity (%kcal: RR = 1.01, 95% CI: 1.00, 1.02, *p* = 0.030; %g: RR = 1.01, 95% CI: 1.00, 1.02, *p* = 0.003) and low HDL-cholesterol (%kcal: RR = 1.02, 95% CI: 1.01, 1.04, *p* = 0.041). No significant associations between UPF consumption and other metabolic syndrome components were observed.
Mendonca et al. (2017) [27]	Spain (SUN cohort)	14,790 36.3% men 63.7% women Age: 36.3 ± 10.3 years	Hypertension	Mean follow-up: 9.1 years	Semi-quantitative FFQ (136 items)	Sex, age, baseline BMI, physical activity, hours of television watching, smoking status, following a special diet at baseline, use of analgesics, alcohol consumption, family history of hypertension, hypercholesterolemia, total energy intake, fruit and vegetable consumption, and olive oil intake.	servings/d	Participants in the third tertile of UPF consumption were at a higher risk of developing hypertension (HR = 1.21, 95% CI: 1.06, 1.37, *P*_trend_ = 0.004) than participants in the first tertile.
Monge et al. (2021) [23]	Mexico (Mexican Teachers’ Cohort)	64934 participants (only women) Age: 41.7 ± 7.2 years	Hypertension	Median follow-up: 2.2 years	Semi-quantitative FFQ (140 items)	Age, smoking status, physical activity, menopausal status, ethnicity, internet access and insurance for serious conditions, family history of hypertension, total energy intake, and multivitamin supplementation.	%UPF_energy_	No association between categories of %UPF_energy_ (≤20%, 21–25%, 26–35%, 36–45% >45% energy/d) and incident hypertension was found. Compared with the first category, IRRs were 0.96 (95% CI: 0.86, 1.07), 0.92 (95% CI: 0.84, 1.02), 0.95 (95% CI: 0.85, 1.06), and 0.98 (95% CI: 0.84, 1.14).
Scaranni et al. (2021) [18]	Brazil (ELSA cohort)	8754 participants 42% men 58% women Median age: 49.0 years	Hypertension	Mean follow-up: 3.9 years	114-item FFQ	Sex, age, self-declared color/ race, education, smoking, alcohol consumption, antihypertensive drug use, Na consumption, physical activity, total daily energy intake, and BMI.	%UPF_energy_	Participants with higher UPF consumption had a marginally significant greater risk of developing hypertension (OR = 1.17; 95% CI: 1.00, 1.37) than participants with lower UPF consumption.
Srour et al. (2019) [22]	France (French NutriNet-Santè cohort)	1047,07 participants 20.8% men 79.2% women Age: 42.7 ± 14.5 years	Type 2 Diabetes	Median follow-up: 6 years	24 h dietary record	Sex, age, BMI, weight change during follow-up, educational level, smoking status, physical activity level, number of 24 h dietary records, alcohol intake, energy intake without alcohol, overall diet quality, family history of diabetes, baseline dyslipidemia and hypertension, and treatments for these conditions.	g/day	An increment of 10% of UPFs in diet was associated with an increased risk of T2D (HR = 1.13, 95% CI: 1.03, 1.23, *p* = 0.04). Similarly, a 100g/day increment in UPF consumption was associated with the risk of T2D (HR = 1.05; 95% CI: 1.02, 1.08, *p* = 0.003).
Duan et al. (2022) [24]	Netherlands (Lifelines cohort)	70,421 participants 41.4% men 58.6% women Age 49.1 ± 8.8 years	Type 2 Diabetes	Median follow-up: 3.4 years	Semi-quantitative FFQ (110 items)	Sex, age, BMI, educational level, energy intake, alcohol intake, Life diet score, smoking status, physical activity, and TV-watching time.	%UPF_intake_	An increment of 10% in UPF consumption was associated with a 25% higher risk of developing T2D (OR = 1.25; 95% CI: 1.16, 1.34).
Levy et al. (2021) [30]	England, Scotland and Wales (UK Biobank)	21,730 participants 47.1% men 52.9% women Age: 55.8 ± 7.4 years	Type 2 Diabetes	Mean follow-up: 5.4 years	24 h dietary recall	Sex, age, BMI, smoking, physical activity level, ethnicity, family history of T2D, Index of Multiple Deprivation (IMD), and total energy intake.	%UPF_intake_	Participants in the highest quartile of UPF consumption were at a higher risk for T2D (HR = 1.44; 95% CI: 1.04, 2.02, *P*_trend_ < 0.028) than participants in the lowest quartile. Moreover, a 10%-point increment in UPF consumption was associated with a 12% increased risk of T2D (HR = 1.12, 95% CI: 1.04, 1.20).
Llavero-Valero et al. (2021) [28]	Spain (SUN cohort)	20,060 participants 38.5% men 61.5% women Age: 37.4 ± 12.2 years	Type 2 Diabetes	Median follow-up: 12 years	Semi-quantitative FFQ (136 items)	Age, sex, BMI, educational level, smoking status, 8-item active + sedentary lifestyle score, following a special diet at baseline, snacking, and family history of diabetes.	g/day	Participants in the highest tertile of UPF consumption were at a higher risk of T2D than participants in the lowest tertile (HR = 1.53, 95% CI: 1.06, 2.22, *P*_trend_ = 0.024). After using repeated measurements of UPF consumption, the association remained significant (HR = 1.65, 95% CI: 1.14, 2.38).
Donat-Vargas et al. (2021) [29]	Spain (ENRICA cohort)	1082 participants 48% men 52% women Age: 68 ± 6 years	Dyslipidemia	5–7 years	Dietary history (DH-ENRICA) record	Sex, age, BMI, smoking status, physical activity, educational level, marital status, total energy intake, alcohol consumption, fiber intake, consumption of unprocessed or minimal processed foods, number of medications, and number of chronic diseases.	%UPF_energy_	Participants in the uppermost tertile of UPF consumption were at a higher risk for incident low HDL cholesterol (OR = 2.23; 95% CI: 1.22, 4.05; *P*_trend_ = 0.012) and hypertriglyceridemia (OR = 2.66, 95% CI: 1.20, 5.90; *P*_trend_ = 0.011) than participants in the lowest tertile. However, the consumption of UPF was not associated with the incident risk of high LDL cholesterol.
Scaranni et al. (2022) [19]	Brazil (ELSA cohort)	5275 participants 42.2% men 57.8% women Age: 50.6 ± 8.8 years	Dyslipidemia	4 years	Semi-quantitative FFQ (114 items)	Sex, age, BMI, schooling, smoking, physical activity, alcohol consumption, total energy intake, diabetes and time since baseline, and Brazilian Healthy Eating Index—Revised (BHEI-R).	g/day	Individuals with medium and high consumption of UPF had higher risks of developing isolated hypertriacylglycerolemia (OR = 1.14, 95% CI: 1.03, 1.26 and OR = 1.30, 95% CI: 1.17, 1.45), isolated hypercholesterolemia (OR = 1.12, 95% CI: 1.00, 1.27 and OR = 1.28, 95% CI: 1.12, 1.47), mixed hyperlipidemia (OR = 1.21, 95% CI: 1.05, 1.39 and OR = 1.38, 95% CI: 1.18, 1.62), and low HDL (OR = 1.12, 95% CI: 1.00, 1.24 and OR = 1.18, 95% CI: 1.05, 1.32), respectively, than participants who consumed less UPF.
